# Changing Burden of Haematolymphoid Tumours in AYA in Poland: Organizational Challenges for the Healthcare System (Current Status and Future Perspectives)

**DOI:** 10.3390/cancers18162721

**Published:** 2026-08-21

**Authors:** Lukasz Taraszkiewicz, Patryk Włodarczyk, Michał Nocek, Maciej Trojanowski, Patrycja Filipek, Urszula Wojciechowska, Joanna A. Didkowska

**Affiliations:** 1 Polish National Cancer Registry, Maria Sklodowska-Curie National Research Institute of Oncology, 02-781 Warsaw, Polandjoanna.didkowska@nio.gov.pl (J.A.D.); 2 Greater Poland Cancer Registry, Greater Poland Cancer Center, 61-866 Poznan, Poland; 3 Polish Onco-Haematology Registry, Maria Sklodowska-Curie National Research Institute of Oncology, 02-781 Warsaw, Poland

**Keywords:** onco-hematology, health service research, population-based cancer registry, AYA, incidence trends, healthcare system organization, access to cancer care, geographical disparities

## Abstract

**Background/Objectives**: Hematological malignancies (HMs) are an important component of the cancer burden in adolescents and young adults (AYA, 15–39 years), requiring long-term specialized care. In Poland, recent healthcare reforms under the National Oncology Network (KSO) have not formally included haemato-oncology or AYA-specific needs. **Methods**: A population-based study was conducted using data from the Polish National Cancer Registry. AYAs diagnosed with HM between 2000 and 2022 were included. Descriptive analyses and projections were based on cases diagnosed between 2000 and 2022, whereas age-specific incidence trend analyses were restricted to the 2015–2022 period. Tumours were classified according to the HAEMCARE framework. Age-standardized incidence rates (ASIRs), annual percentage changes (APCs), and short-term projections through 2028 were estimated using generalized linear models. Additionally, data from the National Health Fund (NFZ) were analyzed to assess the geographical distribution of reimbursed drug programmes dedicated to selected HMs. **Results**: A total of approximately 23,000 AYA patients diagnosed with HMs were identified. Hodgkin lymphomas (HL) were the predominant tumour type across all age groups, while lymphoblastic leukemia/lymphoma was most common among younger AYAs and diffuse large B-cell lymphoma (DLBCL) increased in relative importance with age. Between 2015 and 2022, ASIRs remained stable only in two age groups (20–24 and 25–29), whereas statistically significant increases were observed among individuals aged 15–19, 30–34 and 35–39 years. Despite largely stable incidence patterns, projections indicated a growing absolute number of cases for DLBCL and follicular lymphomas. Analysis of NFZ data revealed substantial regional variation in the availability of reimbursed drug programmes, with up to six-fold differences in facility density between voivodships. **Conclusions**: HM epidemiology among AYAs in Poland is characterized by increasing incidence in selected lymphoma subtypes alongside declining mortality. Incorporating haemato-oncology and AYA-specific needs into national cancer planning frameworks should therefore be considered essential for future healthcare system preparedness.

## Simple Summary

Adolescents and young adults diagnosed with hematological cancer face unique medical and psychosocial challenges that differ from those experienced by children and older adults. Despite the increasing recognition of their specific needs, data on the burden of these diseases and availability of specialized care remain limited in many countries, including Poland. In this study, we examined long-term trends in hematological malignancies among patients aged 15–39 years and the geographical distribution of cancer care services for this patient population across Poland. Our findings show changing patterns of disease occurrence across age groups, a growing number of long-term survivors, and increasing reliance on advanced molecular pathology and targeted therapies. There is a strong concentration of highly specialized treatment services in the few regions of Poland representing the structure of major academic and leading comprehensive cancer centres. These results provide important evidence for healthcare planning and highlight the need for dedicated care pathways that address the clinical, psychological, and long-term follow-up needs of AYA group.

## 1. Introduction

Adolescents and young adults (AYAs), defined as individuals aged 15–39 years, represent a biologically and psychosocially distinct population whose needs should be specifically addressed by the healthcare system. This pragmatic age range was originally adopted by the U.S. National Cancer Institute and subsequently endorsed by international organizations, including European Society for Medical Oncology, to facilitate consistency and comparability across research despite the acknowledged heterogeneity of this population [1,2]. While historically overshadowed by pediatric and older adult cancer research, recent AYA-related studies indicate a concerning rise in the global burden of cancer among this group [3,4]. Although solid tumours, particularly those originating in breast, cervix or thyroid gland account for the majority of incident cancers in this age group, hematolymphoid tumours, predominantly leukemias, remain the primary drivers of cancer-related mortality [5,6].

Epidemiological data reveal a significant geographical survival gap across Europe. While the average five-year relative survival of AYAs diagnosed with cancer is 84%, outcomes in Eastern European countries are generally lower for most cancer sites [7]. Recent studies have also shown that these countries experience significantly higher mortality rates for many AYA-relevant malignancies, when compared with Western and Northern Europe, despite often reporting lower overall incidence rates [8]. Over the last three decades, the absolute number of HMs diagnosed among AYAs has increased, whereas age-standardized mortality rates for leukemias and lymphomas have declined because of therapeutic advances. However, another important gap has been identified, namely the difference in treatment outcomes between AYAs and children diagnosed with the same type of leukaemia [9,10]. Despite these disparities, declining mortality has resulted in a growing population of long-term survivors, leading to increasing overall cancer prevalence and the substantial burden of chronic survivorship care [11]. To address these needs, international recommendations increasingly advocate the use of pediatric-inspired treatment protocols, as well as referrals to specialized expert centres, and age-appropriate multidisciplinary teams capable of addressing the unique holistic needs such as fertility preservation and mental health [1].

In Poland, the National Cancer Network (KSO), established in 2025, aims to standardize oncological care, but HMs and patients under 18 are currently excluded [12,13]. Recognizing the historical neglect of this group, Polish experts have recently co-developed a formal Polish adaptation of the National Comprehensive Cancer Network (NCCN) Guidelines for AYA Oncology. Nevertheless, the biggest concern in Poland is still the lack of clearly established AYA haemato-oncology structures in the public healthcare system [2]. Although the introduction of the fast oncology pathway in 2015 aimed to expedite cancer care in Poland, evidence regarding its impact on AYA patients remains scarce [14]. The current organization of care creates a systemic transition gap, as patients younger than 18 years are managed within pediatric oncology and hematology services, whereas adult care pathways, including fast oncology pathway procedures, apply only to adults. This administrative division does not reflect the biological and psychosocial continuum of the AYA population [15]. Dedicated AYA haemato-oncology pathways remain largely dependent on local initiatives rather than national regulations or dedicated funding mechanisms. Despite the distinct clinical and psychosocial needs of AYA patients with HMs, there is limited evidence on the epidemiological burden of these diseases and the organization of healthcare services for this population in Poland. In particular, little is known about long-term incidence trends, geographical patterns of care provision, and potential gaps in access to age-appropriate services. Addressing these knowledge gaps is important for informing healthcare planning and policy making.

In this study, we analyze epidemiological trends and treatment-related infrastructure relevant to AYA patients with HM in Poland, with particular emphasis on identifying potential gaps between the capabilities of the current public healthcare system and the real-world needs of this patient population.

## 2. Materials and Methods

### 2.1. Data Source and Study Population

Data were obtained from the Polish National Cancer Registry (KRN), a nationwide, general population-based registry collecting information on malignant neoplasms diagnosed in Poland [16]. The study included adolescents and young adults (AYA) aged 15–39 years diagnosed with haematolymphoid tumours between 1 January 2000 and 31 December 2022. Descriptive epidemiological analyses, including case counts, subtype distribution, and temporal trends in the burden of selected HMs were based on the complete study period (2000–2022), whereas age-specific incidence trend analyses were restricted to the 2015–2022 period, representing a more homogeneous healthcare environment following implementation of the fast oncology pathway in 2015.

Cases were identified using morphology codes 9590/3-9989/3 according to the International Classification of Diseases for Oncology, Third Edition (ICD-O-3) [17]. Only malignant neoplasms (behaviour code/3) were included in the analysis. Due to incomplete nationwide registration and limited data comparability before the establishment of the Polish Haemato-Oncological Registry (PROH), myeloproliferative neoplasms and other myeloproliferative diseases were excluded from the final analysis.

Hematological malignancies were grouped according to the HAEMACARE and InterLymph recommendations (Table S1) [18]. The relative distribution of tumour types was evaluated separately for males and females across five AYA age brackets (15–19, 20–24, 25–29, 30–34, 35–39).

Cancer cases identified through death certificates only or autopsy reports were excluded. Time-trend analysis was based on the date of incidence recorded in the registry database. Registration and coding of multiple primary sites followed recommendations of the European Network of Cancer Registries (ENCR) applicable for these tumour types [19].

Healthcare system-related data were derived from the database of publicly funded services maintained by the National Health Fund (NFZ), including information on medical facilities contracted to provide oncology care and drug programme (DP) services for patients with HM [20]. Separate analysis was conducted for DPs dedicated to B-cell lymphomas (BCLs), T-cell lymphomas (TCLs), Hodgkin lymphomas (HLs), acute lymphoblastic leukemias (ALLs), acute myeloid leukemias (AMLs), chronic myeloid leukemias (CMLs), and chronic lymphoblastic leukemias (CLLs).

Population denominators stratified by age, sex, and calendar year were obtained from official demographic statistics provided by Statistics Poland [21].

The characteristics of the study population are presented in Table 1 and Table S2. To facilitate presentation of the results, the five AYA age groups were further aggregated into broader categories: 15–19 years (adolescents), 20–29 years (young adults), and 30–39 years (older AYAs).

### 2.2. Data Quality and Cancer Case Verification

The study dataset underwent a multistep data quality assessment procedure before statistical analysis. Verification included crosschecking of International statistical Classification of Diseases and related health problems, 10th revision (ICD-10) diagnosis and ICD-O morphology code consistency, harmonization of historical coding practices. Duplicate notifications and coding inconsistencies had been resolved during registry quality assurance. Additional validation procedures included a review of selected text-based clinical and pathology-related descriptions extracted from plain-text fields in electronic cancer notifications available in the registry database. Cancer cases with incomplete information preventing reliable classifications were excluded from subtype-specific analysis.

### 2.3. Statistical Analysis

Data on the number of hematological cancer cases in Poland were aggregated for the AYA population (15–39 years). To initially assess their temporal structure, the data were visualized using a stacked area chart. To evaluate these temporal patterns formally, the burden of disease was analyzed using three distinct epidemiological measures: absolute case counts, age-specific incidence rates (calculated separately for five-year age groups: 15–19, 20–24, 25–29, 30–34 and 35–39 years per 100,000 population), and truncated age-standardized incidence rates (TASRs) per 100,000 population for the overall AYA cohort. TASRs were calculated via the direct method for the truncated age range of 15–39 years, applying the specific age-bracket weights extracted from the Segi-Doll World Standard Population to rigidly control for shifting internal demographic structures within the AYA cohort over the study period. Joinpoint (JP) regression analysis employing Monte Carlo permutation tests was performed to identify potential trend breakpoints across all three metrics: absolute case counts, age-specific rates and overall TASRs [22]. Given the limited number of time points available for the segment (2015–2022 for the JP analysis), the models were constrained to allow a maximum of one potential breakpoint.

To determine the long-term incidence trends and calculate the Annual Percentage Change (APC) for specific disease categories across the extended timeline (2000–2023), Generalized Linear Models (GLMs) were utilized. Prior to final model selection, the assumption of equidistribution was formally evaluated using the Cameron-Trivedi test [23]. Due to the confirmation of significant overdispersion in the count data (*p* < 0.05), the classic Poisson model was rejected in favour of a quasi-Poisson (QP) framework to prevent the underestimation of standard errors. The GLM with a log link function was specified as:

$$\log\left(E\left[Y_{it}\right]\right)=\beta_{0}+\beta_{1}\cdot {Year}_{t}+offset(\log\left({Population}_{it}\right))$$

where $Y_{it}$ represents the observed number of incident cases for disease category i in year t, $E\left[Y_{it}\right]=\mu_{it}$ is the expected count, and the variance is modelled as $Var\left(Y_{it}\right)={\phi \mu }_{it}$, with ϕ denoting the estimated overdispersion parameter. The natural logarithm of the annual age-specific population size was incorporated as an offset variable to accurately estimate the underlying biological risk trend, modelling incidence rates directly and independently of demographic fluctuations. The APC was derived from the continuous year coefficient $\left(\beta_{1}\right)$ using the following formulation:

$$APC=\left(e^{\beta_{1}}-1\right)\cdot 100\%$$

The 95% confidence intervals (CIs) for the APC were explicitly computed using the Wald-type standard errors (SE) of the $\beta_{1}$ coefficient, according to the formula:

$$95\%\;CI=\left(e^{\beta_{1}\pm 1.96\cdot SE}-1\right)\cdot 100\%.$$

The fitted QP models were then employed to forecast the number of haematological cancer burden in the AYA cohort for the period 2023–2028. Official demographic data for Poland were available up to 2025; therefore, precise, two-stage forecasting architecture was implemented. In the first phase (2023–2025), the predictive models utilized the official actual demographic projections provided by Statistics Poland (GUS) as the denominator (baseline offset) to calculate the projected absolute number of incident cases. In the second phase (2026–2028), the age-specific population size for 2026–2028 was linearly extrapolated based on historical demographic trajectory from 2015 to 2025. This extrapolated population served as the baseline offset for the GLM to generate case projections up to 2028. Uncertainty intervals for the projected case counts and rates (2023–2028) were expressed as 95% prediction intervals (PIs). These intervals account for the statistical uncertainty in the model parameters (${\hat{\beta }}_{0},{\hat{\beta }}_{1}$) and were calculated on the link (log) scale using standard errors of the linear predictor:

$${\mathrm{PI}}_{\mathrm{link}}={\hat{\eta }}_{t}\pm z_{\alpha /2}\cdot \mathrm{SE}\left({\hat{\eta }}_{t}\right)$$

where ${\hat{\eta }}_{t}$ is the predicted log-incidence rate. The intervals were subsequently transformed back to the response scale using the exponential function:

$${\mathrm{PI}}_{\mathrm{response}}=\exp\left({\mathrm{PI}}_{\mathrm{link}}\right)$$

Finally, to facilitate clinical and epidemiological interpretation, both historical and projected case counts were converted into crude incidence rates per 1,000,000 inhabitants.

All statistical analysis were performed using the R statistical environment (R language R-4.5.2; RStudio 2026.01.1 Build 403) and Joinpoint Trend Analysis Software (Version [6.0.1]). A two-tailed *p*-value of <0.05 was considered statistically significant.

### 2.4. Geographical Visualizations

Geographic visualizations and map preparation were performed using the open-source Geographic Information System (GIS) software QGIS LTR 3.40.4-Bratislava (QGIS Development Team). QGIS is a free and open-source GIS platform developed and maintained by the Open Source Geospatial Foundation (OSGeo) [24]. For each programme category, all facilities listed by the NFZ as eligible to provide treatment were identified and assigned to the corresponding city and voivodship. When a single DP was available within both outpatient specialist care (AOS) and inpatient hospital services in the same medical facility, the programme was counted only once to avoid duplication of treatment locations.

To facilitate comparison between regions with different population sizes, the number of programme locations was standardized per 1,000,000 AYAs population. To ensure consistency between the eligible population and the analyzed healthcare services, geographical analyses were standardized to the population aged 18–39 years, as the vast majority of the NFZ drug programmes are approved exclusively for adult patients (≥18 years). Population denominators were derived from official 2025 demographic estimates published by Statistics Poland (GUS). Maps were generated in Microsoft Excel using its built-in map chart tool, which relies on OpenStreetMap geographic data.

### 2.5. Ethical Considerations

In Poland, reporting of malignant tumours to the KRN is mandatory according to national legislation. The present study was based exclusively on aggregated registry data collected routinely within statutory activities of cancer registration system. No directly identifiable patient information was used. According to applicable national regulations, studies based solely on aggregated and anonymous registry data do not require individual informed consent or formal approval by a bioethics committee. A detailed description of the organization, legal framework, data collection procedures of the KRN has been published elsewhere [25,26]. This study has been conducted following the Strengthening the Reporting of Observational Studies in Epidemiology (STROBE) guidelines. It is important to note that no direct patient involvement was needed in this study.

## 3. Results

### 3.1. Distribution of Haematolymphoid Tumours by Age and Sex

**Figure 1:**
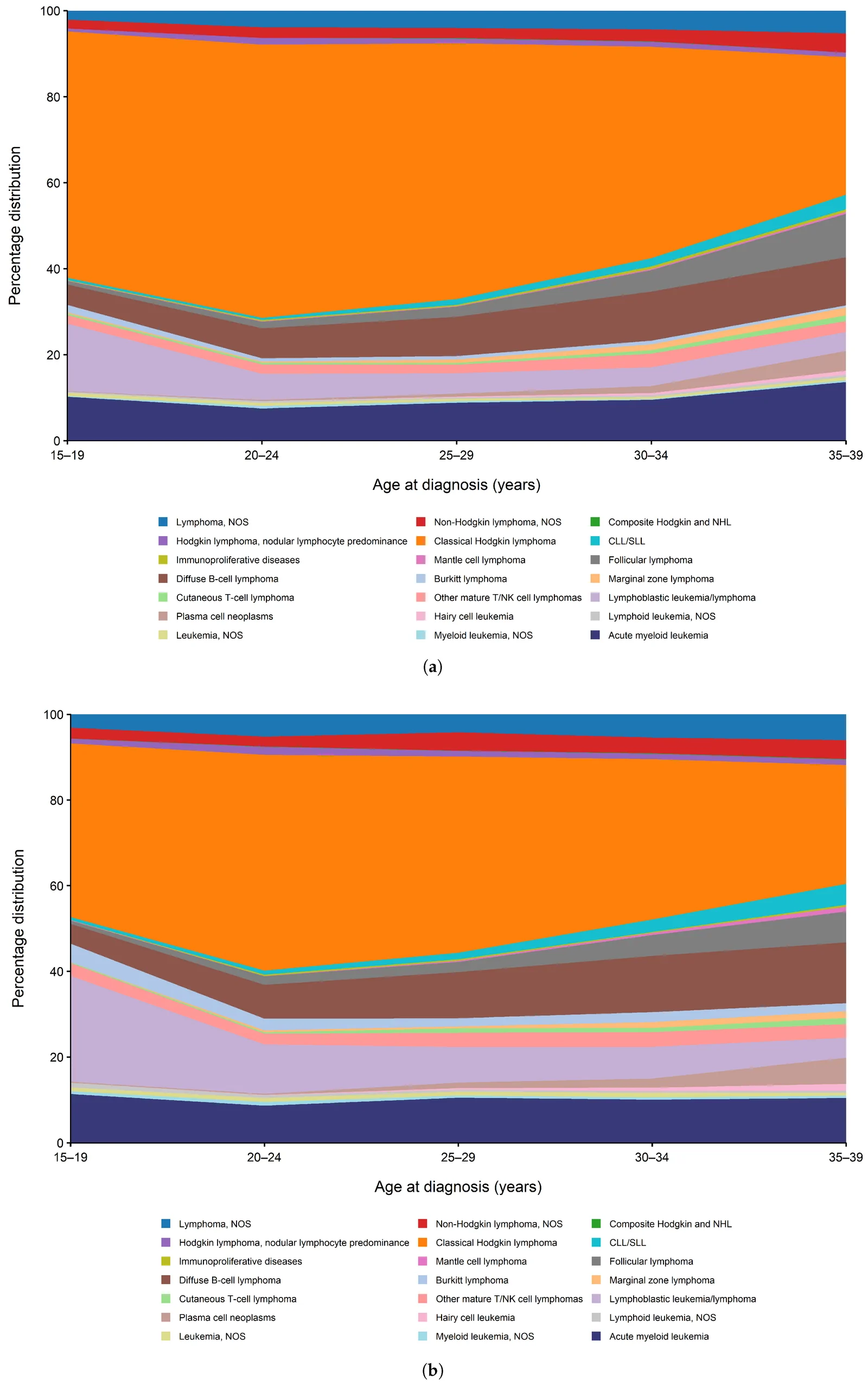
Sex-specific percentage distribution of hematological malignancies diagnosed in Poland between 2000 and 2022 according to five AYA age groups (15–19, 20–24, 25–29, 30–34, and 35–39 years): (**a**) females; (**b**) males. Percentages were calculated within each age group and sex category.

The distribution of the total number of HM cases registered in KRN between 2000 and 2022 varied considerably across AYA age groups and between sexes (Figure 1): classical HL represented the most frequent diagnostic category in both females and males throughout the entire AYA age spectrum. The highest proportion was observed in the 20–24 age group, where cHL accounted for 63.51% of all HMs among females and 50.36% among males. Thereafter, its relative contribution gradually declined with increasing age, reaching 31.99% and 27.72%, respectively, among individuals aged 35–39 years. Lymphoblastic leukemia/lymphoma (LL) was the second most common malignancy in the youngest AYA group (15–19 years), accounting for 15.68% of diagnoses among females and 24.64% among males. However, its relative contribution declined markedly with age, falling to approx. 4–5% in the 35–39 age group. In contrast, diffuse large B-cell lymphoma (DLBCL) demonstrated the opposite pattern, increasing steadily from approx. 5% of diagnoses in the youngest age group to 11.19% among females and 14.19% among males aged 35–39 years.

Acute myeloid leukemia (AML) was consistently represented across all age groups in both sexes, accounting for approx. 8–14% of diagnoses, with the highest proportion observed among females aged 35–39 years (13.63%). Overall, the results demonstrate a shift from lymphoblastic and Hodgkin-type malignancies in younger AYAs towards mature B-cell neoplasms and myeloid tumours in older age groups.

### 3.2. Age-Specific Incidence Trends of HMs

**Figure 2:**
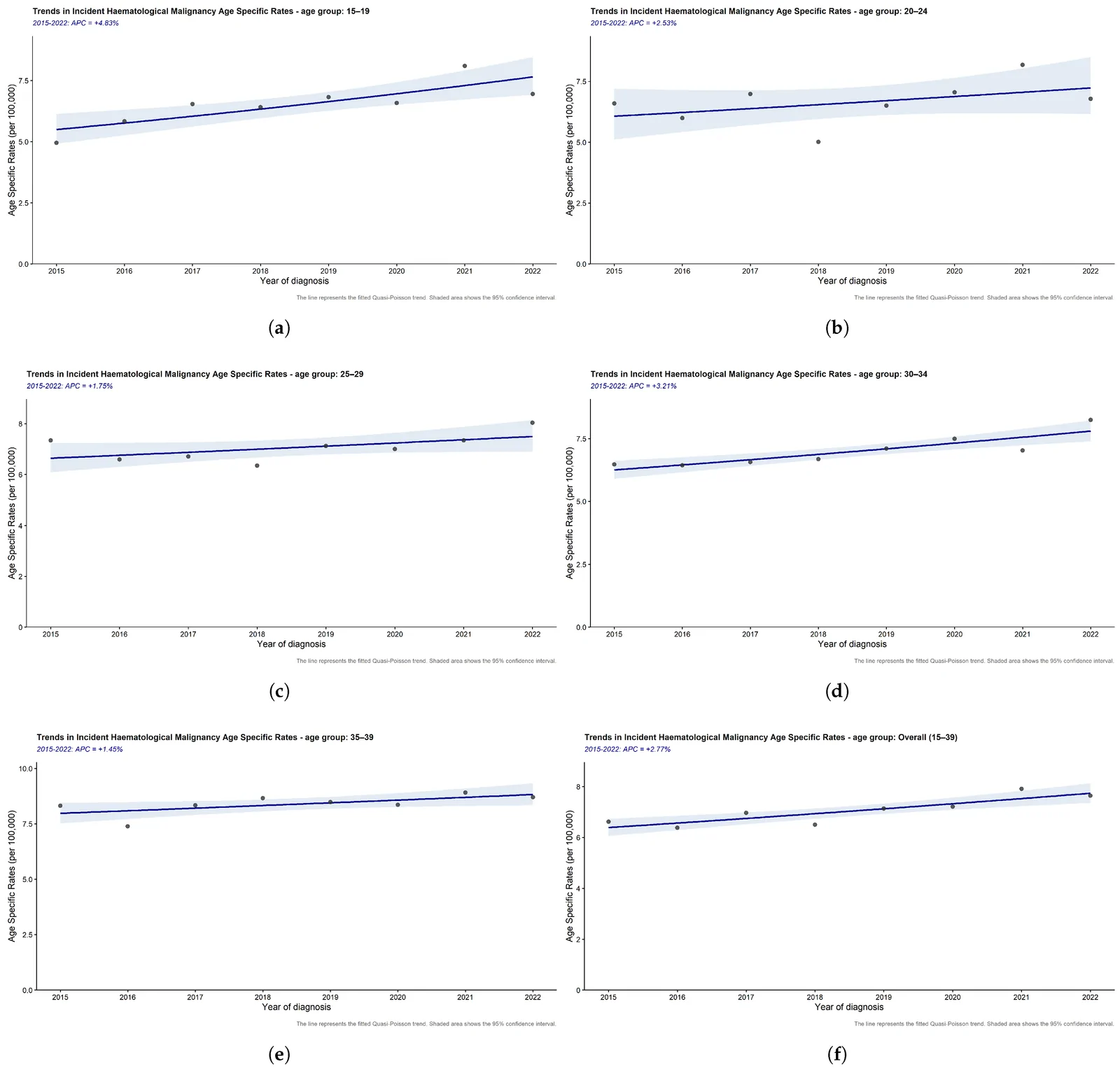
Long-term incidence trends of HM among AYA in Poland between 2015 and 2022, stratified by age group. Panels correspond to the following age groups: (**a**) 15–19 years; (**b**) 20–24 years; (**c**) 25–29 years; (**d**) 30–34 years; (**e**) 35–39 years; (**f**) 15–39 years; Age Specific Rates (**a**–**e**), Truncated Age Standardized Incidence Rates (**f**).

Age-specific incidence trends of HMs varied across the AYA population. A statistically significant increase was observed among age groups with the highest annual percentage change (APC): 15–19 years (APC = 4.83%; 95% CI: 2.21 to 7.53), 30–34 years (APC = 3.21%; 95% CI: 1.85 to 4.59) and 35–39 years (APC = 1.45%; 95% CI: 0.07 to 2.85). Incidence trends remained relatively stable among individuals aged 20–24 years (APC = 2.53%; 95% CI: −1.46 to 6.68) and 25–29 years (APC = 1.75%; 95% CI: −0.29 to 3.83), as the corresponding confidence intervals included the null value. The trends are presented in Figure 2.

### 3.3. Temporal Trends and Short-Term Projected Burden for Selected Types of HM

**Figure 3:**
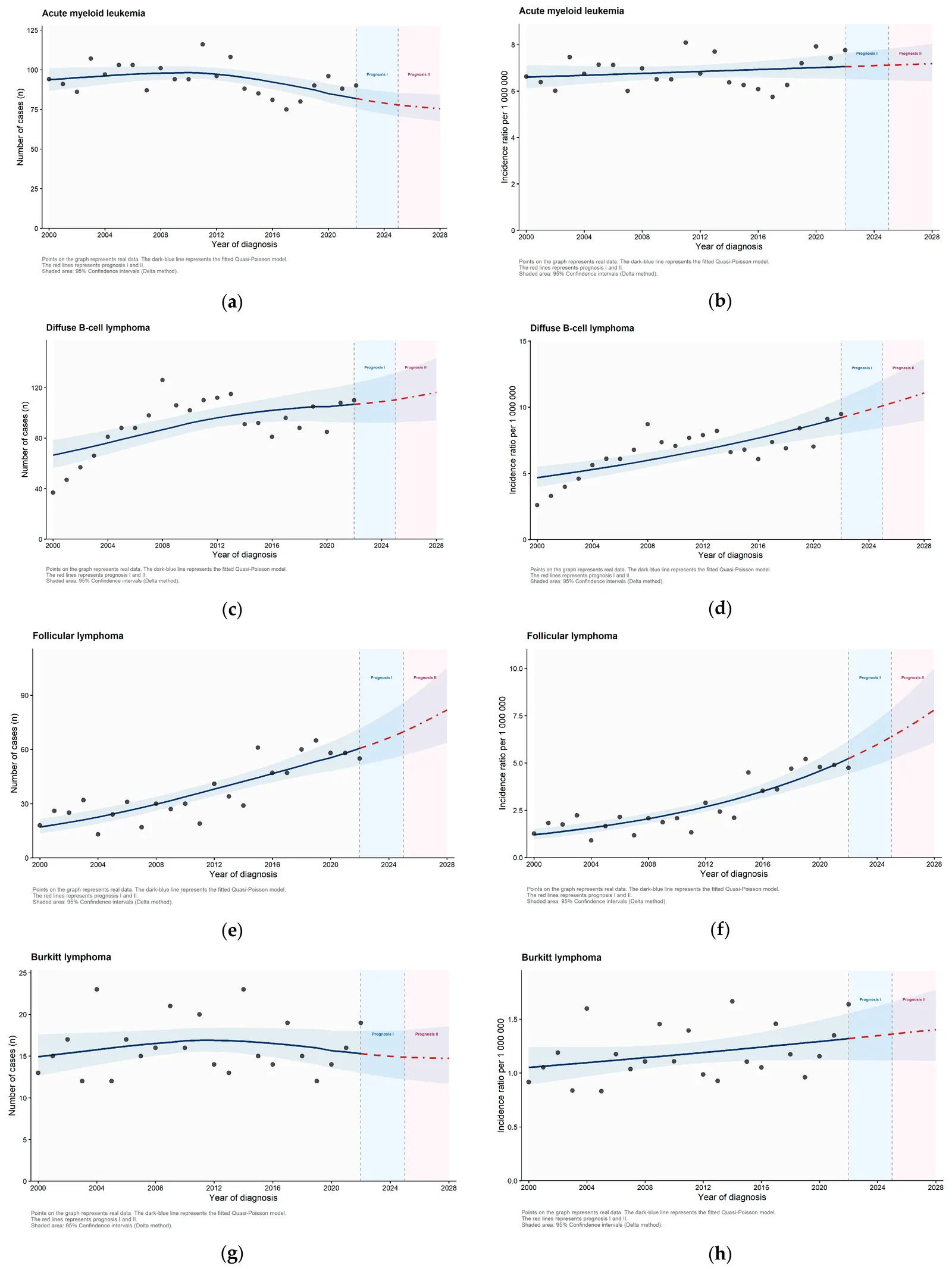

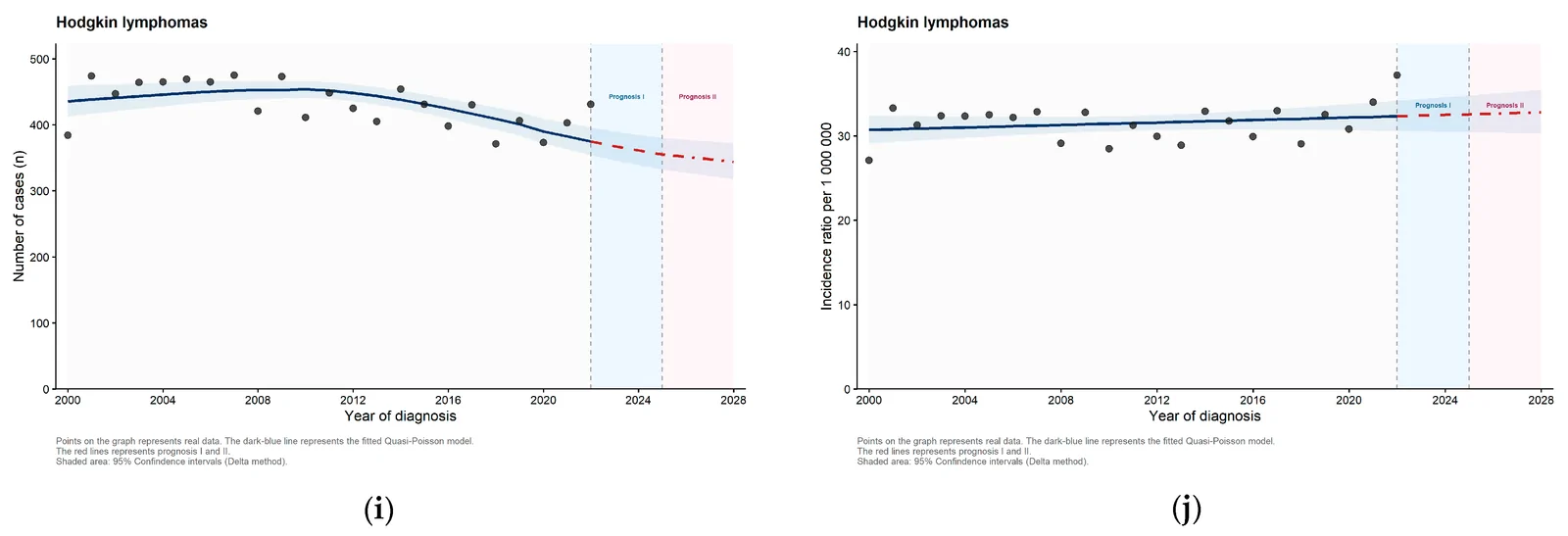
Historical trends and projected burden of selected HMs among AYAs in Poland. For each tumour type the left panel presents the annual number of incident cases, whereas the right panel presents crude incidence rates per 1,000,000 population. Observed values are shown for the period 2000–2022, and projections are presented for 2023–2028. Tumour types included: (**a**,**b**) AML; (**c**,**d**) DLBCL; (**e**,**f**) follicular lymphoma (FL); (**g**,**h**) Burkitt lymphoma; and (**i**,**j**) Hodgkin lymphoma.

Historical and projected trends in AYA differed substantially between tumour types (Figure 3). FL demonstrated the highest projected increase (APC = 6.91%; 95% CI: 5.24–8.60), followed by DLBCL (APC = 3.12%; 95% CI: 1.87–4.38). In contrast, Burkitt lymphoma remained relatively rare, with stable annual case numbers and incidence rates over time (APC = 1.03%; 95% CI: −0.26–2.34).

Hodgkin lymphoma showed a stable trend in crude incidence rates (APC = 0.23%; 95% CI: −0.19–0.66), indicating that demographic changes may contribute to the observed reduction in the absolute number of cases. A similar pattern was observed for AML, which showed no statistically significant temporal trend (APC = 0.3%; 95% CI: −0.31 to 0.91).

### 3.4. Drug Programmes Coverage (Regional Distribution of Medical Facilities)

**Figure 4:**
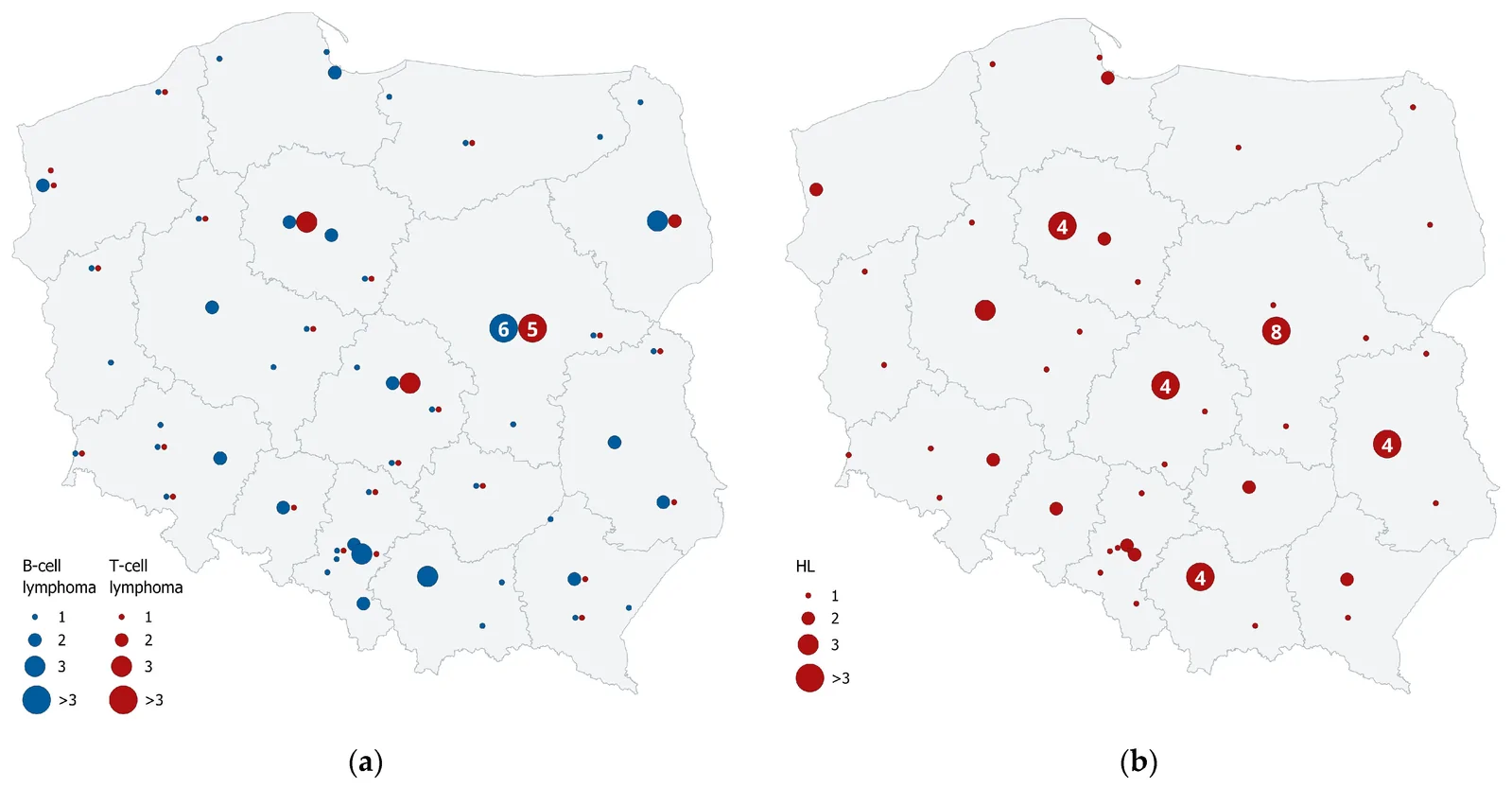

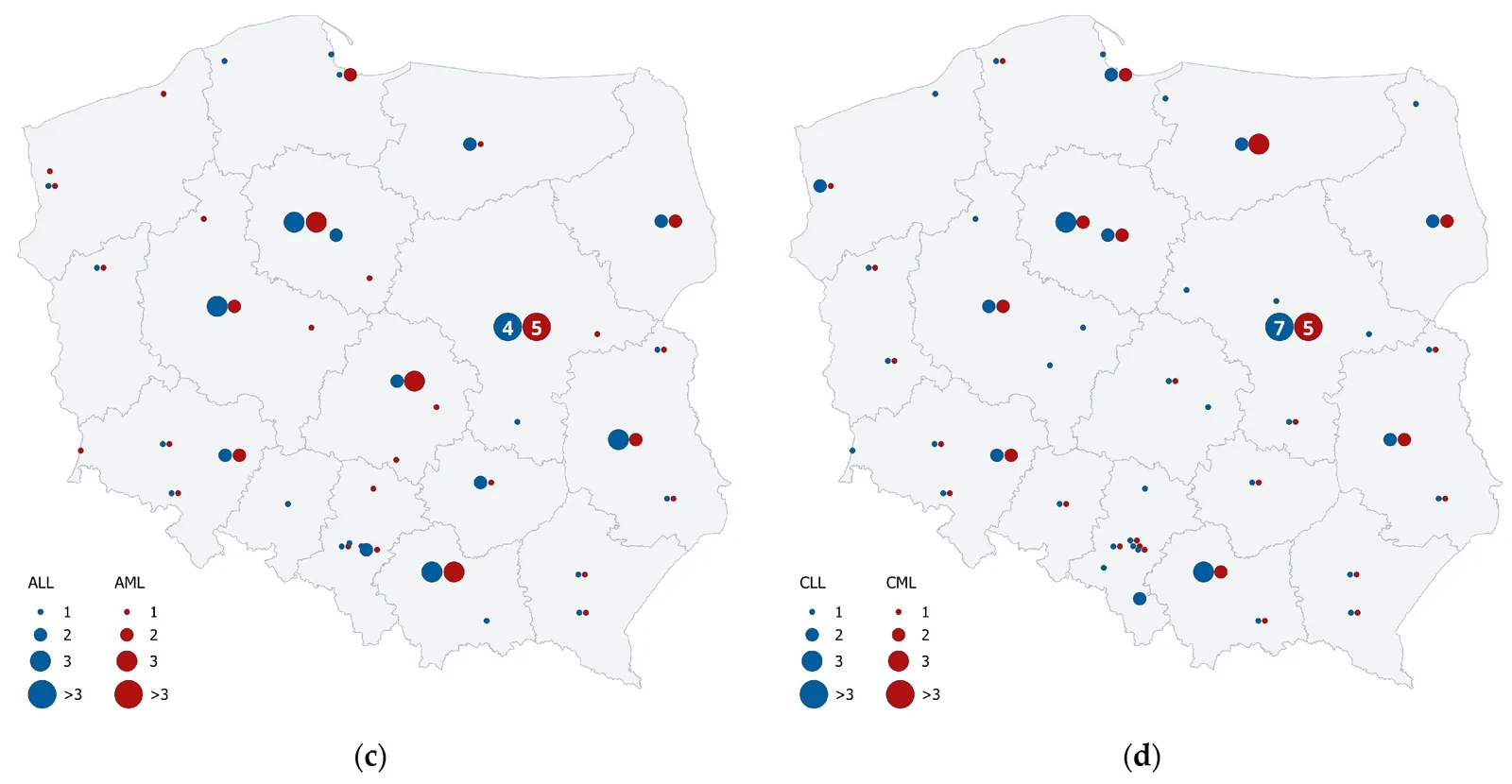
Distribution of medical facilities contracted by the NFZ to provide reimbursed drug programmes for selected hematological malignancies across Polish cities in 2026. Bubble size corresponds to the number of contracted facilities in each city. For cities with more than three contracted facilities, the exact number of facilities is indicated within the bubble. Panels present programmes dedicated to: (**a**) B-cell and T-cell lymphomas; (**b**) HLs; (**c**) ALL and AML; (**d**) CLL and CML.

According to the list of drug programmes reimbursed by the NFZ, several treatment pathways relevant to AYA patients with HMs were available in Poland in 2026, subject to fulfilment of programme-specific eligibility criteria. These programmes covered, among others, HLs, BCLs (incl. DLBCL, MCL, MZL, FL, and PBCL), TCLs, acute leukemias (AML and ALL), chronic leukemias (CLL and CML). As presented in Figure 4, medical facilities contracted by the NFZ and authorized to provide these therapies were predominantly located in the largest cities of individual voivodships. Most of these facilities represented highly specialized oncology, hematology, university, or transplantation centres, although some therapies were also available in smaller regional hospitals and non-academic centres. The observed distribution suggests a substantial degree of centralization of advanced haemato-oncology care within the Polish healthcare system.

**Figure 5:**
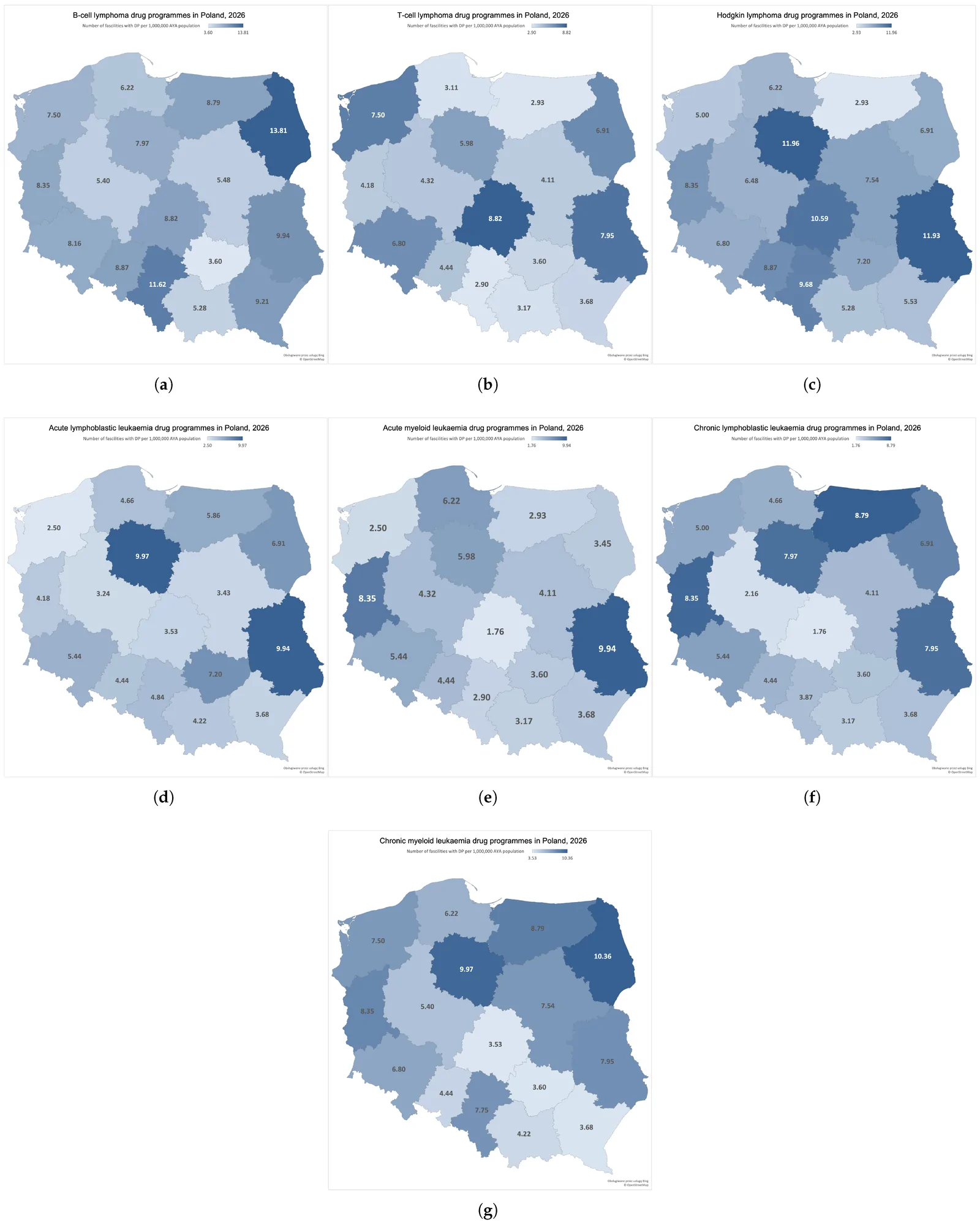
Regional distribution of medical facilities contracted by the NFZ to provide reimbursed drug programmes for selected hematological malignancies across the 16 voivodeships of Poland in 2026, standardized per 1,000,000 AYA population (aged 18–39 years). Panels present programmes dedicated to: (**a**) BCL; (**b**) TCL; (**c**) HL; (**d**) ALL; (**e**) AML; (**f**) CLL; and (**g**) CML.

After standardization for the size of the AYA population, substantial differences between voivodships remained visible (Figure 5). The density of contracted facilities differed considerably between regions and varied according to the type of HM. The highest density was observed in Podlaskie for BCL and CML programmes, in Łódzkie for TCL programmes, in Kujawsko-Pomorskie for HL programmes, and in Warmińsko-Mazurskie for CLL programmes. In contrast, the lowest densities were most frequently recorded in Łódzkie. Regional variation ranged from approximately three-fold for TCL and CML programmes to nearly six-fold for AML programmes, for which the highest density of contracted facilities was observed in Lubelskie (9.94 per 1,000,000 AYA population), and the lowest was recorded in Łódzkie (1.76 per 1,000,000 AYA population).

## 4. Discussion

### 4.1. Changing Epidemiology of HMs in Polish AYA

The present study provides the first comprehensive population-based analysis of hematological malignancies among Polish AYAs. The observed age- and sex-specific distributions were broadly consistent with previous European studies, with Hodgkin lymphoma representing the dominant malignancy across most AYA age groups, while lymphoblastic tumours were more dominant among younger AYAs and mature B-cell neoplasms became increasingly frequent with age [27].

Moreover, the analysis of age-standardized incidence trends across the AYA population between 2015 and 2022 demonstrated increasing incidence rates in the 15–19, 30–34, and 35–39 age groups. These findings support the hypothesis that the AYA population constitutes a biologically heterogeneous group positioned between pediatric and older adult oncology and are consistent with patterns observed in Western European countries, including the Netherlands [28].

### 4.2. Future Burden and Long-Term Follow-Up Challenges

The projected increase in selected mature B-cell neoplasms observed in this study may continue to generate healthcare demand over the coming years. This trend is expected to increase the number of long-term survivors requiring monitoring for treatment-related complications, fertility preservation, psychosocial support, and management of late effects of treatment. The findings from EUROCARE and CONCORD-3 studies suggest that survival gains have not been equally distributed across age groups, with AYAs frequently demonstrating poorer outcomes than children diagnosed with the same tumour type [9,10,29].

### 4.3. Increasing Diagnostic and Therapeutic Complexity

The growing burden of mature B-cell neoplasms has important implications for public healthcare organization. Contemporary haemato-oncology relies more on molecular, cytogenetic, immunophenotypic, and advanced diagnostic pathology to establish an accurate diagnosis and determine eligibility for targeted treatment [30]. Many of the currently reimbursed drug programmes in Poland require molecular confirmation of genetic hallmarks of tumour subtype or biomarker status, increasing demand for highly specialized laboratory infrastructure and expert interpretation. Poland faces severe capacity constraints regarding both advanced diagnostic facilities and the specialized workforce required to handle the expanding workload.

### 4.4. Organization of Haemato-Oncology Care in Poland

The present analysis showed substantial geographical variation in the distribution of facilities contracted to provide a reimbursed drug programme. Even after standardization for the AYA population size, regional differences of up to six-fold were observed for selected programme categories. However, these findings should not be interpreted as direct measures of patient access, as patients may receive treatment outside their region of residence [31]. Nevertheless, travel distance to diagnostic and treatment facilities could negatively affect patients’ quality of life [32,33]. Furthermore, advanced cellular therapies remain highly centralized, with 15 centres authorized to administer CAR-T therapy in Poland in 2025 [34]. Similar patterns of concentration of highly specialized services have been reported across Europe and reflect the increasing complexity of haemato-oncology care [35,36].

An important consideration is that analyzed drug programmes in Poland have not been designed specifically for AYA patients. In most cases, eligibility criteria are determined by disease characteristics and age, with reimbursed programmes primarily intended for adult patients aged 18 years or older. Consequently, younger adolescents diagnosed with comparable HMs are typically managed within pediatric diagnostic and treatment pathways. This administrative separation reflects the broader organizational structure of oncology care in Poland and may contribute to the transition gap frequently described in the AYA literature [5,37,38,39].

Future studies incorporating patient-level treatment data could help evaluate whether geographical differences in service location translate into differences in healthcare utilization, travel burden, or treatment outcomes among AYA patients.

### 4.5. The AYA Gap in Polish Healthcare System

Despite growing international recognition of AYA oncology as a distinct field, dedicated AYA pathways remain limited in Poland. Unlike several Western European countries, the Polish public healthcare system continues to separate pediatric and adult oncology services, creating a transition gap that may affect continuity of care, particularly for patients who require long-term follow up or experience disease relapse during transition from pediatric to adult services. In the absence of dedicated national AYA pathways, decisions regarding the treatment setting for relapse are typically individualized and depend on disease characteristics, previous treatment protocols, available expertise, and local organizational arrangements. Rather than recommending management based solely on chronological age, the recent literature emphasizes coordinated multidisciplinary decision-making involving both pediatric and adult haemato-oncology teams [39,40]. In contrast to countries with dedicated AYA cancer services like the United Kingdom, France or Germany, Polish legislation does not define specific requirements regarding the structure, staffing, or financing of AYA-focused care [41,42,43,44,45]. While the establishment of the KSO represents an important step towards the standardization of cancer care in Poland, hematological malignancies and patients younger than 18 years are not fully incorporated into the current organizational framework. Nevertheless, recent organizational developments indicate increasing recognition of unmet needs within haemato-oncology. In May 2026, a pilot programme of the National Haematology Network (KSH) was launched with the aim of improving coordination and standardization of hematological care [46]. At the same time, dedicated AYA initiatives have been established within the National Research Institute of Oncology in Warsaw and Gliwice, including a multidisciplinary Working Group that aims to identify unmet needs of AYA patients and develop recommendations for future service organization. However, the long-term impact of these initiatives remains uncertain and nationwide implementation of dedicated AYA pathways has yet to be achieved.

### 4.6. Cancer Registration, Data Quality, and Interoperability Challenges

The increasing diagnostic and therapeutic complexities also pose challenges for population-based cancer registration. The establishment of the PROH in 2023 introduced a disease-specific registration based on structured electronic clinical documentation, enabling the collection of more detailed and relevant data [47]. However, our analysis of NFZ drug programmes identified the continued use of selected outdated ICD-10 codes within reimbursement criteria [48]. Such discrepancies between reimbursement systems and contemporary disease classifications may create interoperability challenges between healthcare providers, pathology laboratories, reimbursement databases, and cancer registries, potentially affecting data harmonization and the comparability of epidemiological analyses [49,50,51].

### 4.7. Study Limitations

Our study has several limitations that should be considered when interpreting the findings. Although the analyses were based on nationwide population-based registry data, age-specific trends were modelled using data from 2015 to 2022 to reflect the contemporary organization of cancer diagnosis and reporting following implementation of the fast oncology pathway. The relatively short observation period increases the uncertainty of estimated annual percentage changes and short-term projections, which should therefore be interpreted with appropriate caution.

Secondly, myeloproliferative neoplasms and related disorders were excluded because historical registration before implementation of the PROH was not sufficiently complete and comparable across the study period. Consequently, the presented estimates describe the burden of malignant HMs included under the study definition rather than the full spectrum of hematological neoplasms.

Moreover, the geographical analyses describe the distribution of medical facilities contracted to the NFZ to provide reimbursed drug programmes rather than direct access to care. These analyses do not account for patient mobility, referral pathways, travel distance, waiting times, treatment volume, or eligibility criteria, all of which may influence the actual accessibility of specialized services.

Finally, the present study focused on incidence patterns and organizational aspects of healthcare delivery. Population-based survival and prevalence analyses could not be performed because sufficiently complete and validated vital status information was not yet available within the analytical dataset. These analyses, together with studies evaluating treatment pathways, healthcare utilization, and outcomes across the transition from pediatric to adult haemato-oncology services, represent important directions for future research.

## 5. Conclusions

Future healthcare planning for AYA patients should also take demographic trends into account. After 2025, the AYA population in Poland is expected to decline rather than increase, reflecting the ageing of relatively large birth cohorts out of the 15–39-year age range and their replacement by smaller younger cohorts. Consequently, any stable or increasing number of hematolymphoid tumour cases in this age group would indicate a growing disease burden despite a shrinking underlying population at risk. Given the distinct clinical and psychosocial needs of AYA patients, continuous monitoring of demographic and epidemiological trends will be essential for ensuring adequate resource allocation, workforce planning, and the development of dedicated care pathways within the Polish healthcare system.

The centralized organization of healthcare reimbursement in Poland, with the NFZ acting as the single public payer, creates an opportunity to introduce nationwide system-level changes based on unified guidelines and standardized reporting requirements. This centralized framework is further supported by strategic initiatives such as the NSO and the KSO, which aim to improve the quality, coordination, and standardization of cancer care across the country. These initiatives may facilitate the implementation of harmonized diagnostic pathways, reporting standards, and quality assurance measures for patients with HM, including the AYA population.

However, the reliance on ICD-10 as the primary classification system for reimbursement may limit the granularity of coding for hematological malignancies. Since ICD-10 does not fully capture the diagnostic detail provided by ICD-O-3, discrepancies between clinical, pathological, and reimbursement classifications may compromise data quality in healthcare institutions and contribute to under-registration or misclassification of selected HM subgroups. Improving the alignment between reimbursement and cancer registration systems could therefore enhance the accuracy of epidemiological surveillance and healthcare planning.

## Figures and Tables

**Table 1:** Descriptive statistics of hematological malignancy cases by tumour type, period of diagnosis, sex and median age at the time of diagnosis.

	Characteristic								
	Sex, *n* (*%*)		Age Group, *n* (*%*) *			Median Age	Period of Diagnosis, *n* (*%*)		
Tumour Type	Male	Female	15–19	20–29	30–39	Years	2000–2007	2008–2014	2015–2022
All Cases (*N* = 23,087)	12,135 (53)	10,952 (47)	3413 (15)	9105 (39)	10,569 (46)	28	7837 (34)	7063 (31)	8187 (35)
Leukemia (*n* = 2631)	1444 (55)	1187 (45)	423 (16)	931 (35)	1277 (49)	29	977 (37)	846 (32)	808 (31)
Hodgkin Lymphoma (*n* = 9923)	4565 (46)	5358 (54)	1619 (16)	4787 (48)	3517 (35)	27	3643 (37)	3037 (31)	3243 (33)
Non-Hodgkin Lymphoma (*n* = 5024)	2868 (57)	2156 (43)	460 (9)	1625 (32)	2939 (58)	31	1302 (26)	1619 (32)	2103 (42)
Other Myeloid/Lymphoid (*n* = 2247)	2459 (59)	1682 (41)	818 (20)	1338 (32)	1985 (48)	29	1264 (31)	1205 (29)	1672 (40)
Plasma cell neoplasms (*n* = 413)	253 (61)	160 (39)	6 (1)	56 (14)	351 (85)	36	124 (30)	130 (31)	159 (38)

* Age groups were aggregated into three categories for presentation purposes: 15–19 (adolescents), 20–29 (young adults), and 30–39 (older AYAs).

## Data Availability

Data available on request from corresponding author.

## Supplementary Materials

The following supporting information can be downloaded at: https://www.mdpi.com/article/10.3390/cancers18162721/s1, Table S1: Mapping of ICD-O-3 morphology codes and descriptions to haematological malignancy groups applied in the study, according to the HAEMACARE and the InterLymph recommendations; Table S2: Distribution of haematological malignancies diagnosed among adolescents and young adults (AYAs) in Poland between 2000 and 2022, by tumour type, subtype, and sex.

## Abbreviations

The following abbreviations are used in this manuscript:

KRN	Krajowy Rejestr Nowotworów (Polish National Cancer Registry)
DiLO	Diagnostyka i Leczenie Onkologiczne (Diagnostic and Oncology Treatment)
PROH	Polski Rejestr Onko-Hematologiczny (Polish Onco-hematological Registry)
NFZ	Narodowy Fundusz Zdrowia (National Health Fund)
KSO	Krajowa Sieć Onkologiczna (National Oncological Network)
KSH	Krajowa Sieć Hematologiczna (National Haematological Network)
NSO	Narodowa Strategia Onkologiczna (National Oncological Strategy)
ICD-O-3	International Classification of Diseases for Oncology, Third Edition
ICD-10	International statistical Classification of Diseases and related health problems, 10th revision
AML	Acute Myeloid Leukemia
ALL	Acute Lymphoblastic Leukemia
CML	Chronic Myeloid Leukemia
CLL	Chronic Lymphoblastic Leukemia
HM	Hematological malignancy
AYA	Adolescents and young adults
DLBCL	Diffuse Large B-cell Lymphoma
LL	Lymphoblastic leukemia/lymphoma
NCCN	National Comprehensive Cancer Network
BCL	B-cell Lymphoma
TCL	T-cell Lymphoma
DP	Drug Programme
AOS	Ambulatoryjna Opieka Specjalistyczna (outpatient specialist care)
