# Supplementary material for: Changing Burden of Haematolymphoid Tumours in AYA in Poland: Organizational Challenges for the Healthcare System (Current Status and Future Perspectives)
Source: Cancers (Basel). 2026 Aug 21;18(16):2721. doi: 10.3390/cancers18162721 (PMC13510447; doi:10.3390/cancers18162721)
Supplement: Supplementary file 1 [file cancers-18-02721-s001.zip › cancers-4405216-supplementary.pdf]

Supplementary Table S1. Mapping of ICD-O-3 morphology codes and descriptions to haematological malignancy groups applied in the study, according to the HAEMACARE and the InterLymph recommendations.

Group label	ICD-O-3 morphology code	ICD-O-3 morphology description
Lymphoma, NOS	95903	Malignant lymphoma, NOS
Non-Hodgkin lymphoma, NOS	95913	Malignant lymphoma, non-Hodgkin, NOS
Composite Hodgkin and NHL	95963	Composite Hodgkin and non-Hodgkin lymphoma
Hodgkin lymphoma, nodular lymphocyte predominance	96593	Nodular lymphocyte predominant HL
Classical Hodgkin lymphoma	96503	HL, NOS
Classical Hodgkin lymphoma	96513	HL, lymphocyte-rich
Classical Hodgkin lymphoma	96523	HL, mixed cellularity
Classical Hodgkin lymphoma	96533	HL, lymphocyte depletion
Classical Hodgkin lymphoma	96543	HL, lymphocyte depletion, diffuse fibrosis
Classical Hodgkin lymphoma	96553	HL, lymphocyte depletion, reticular
Classical Hodgkin lymphoma	96613	Hodgkin granuloma [obs]
Classical Hodgkin lymphoma	96623	Hodgkin sarcoma [obs]
Classical Hodgkin lymphoma	96633	HL, nodular sclerosis, NOS
Classical Hodgkin lymphoma	96643	HL, nodular sclerosis, cellular phase
Classical Hodgkin lymphoma	96653	HL, nodular sclerosis, grade 1
Classical Hodgkin lymphoma	96673	HL, nodular sclerosis, grade 2
CLL/SLL	96703	Small B lymphocytic lymphoma
CLL/SLL	98233	Chronic lymphocytic leukemia
Immunoproliferative diseases	97603	Immunoproliferative disease, NOS
Immunoproliferative diseases	96713	Lymphoplasmacytic lymphoma
Immunoproliferative diseases	97613	Waldenström macroglobulinemia
Immunoproliferative diseases	97623	Heavy chain disease, NOS
Mantle cell lymphoma	96733	Mantle cell lymphoma
Follicular lymphoma	96903	Follicular lymphoma, NOS
Follicular lymphoma	96913	Follicular lymphoma, grade 2
Follicular lymphoma	96953	Follicular lymphoma, grade 1
Follicular lymphoma	96983	Follicular lymphoma, grade 3
Diffuse B-cell lymphoma	96753	Diffuse mixed small and large cell [obs]
Diffuse B-cell lymphoma	96783	Primary effusion lymphoma
Diffuse B-cell lymphoma	96793	Mediastinal large B-cell lymphoma
Diffuse B-cell lymphoma	96803	Diffuse large B-cell lymphoma, NOS
Diffuse B-cell lymphoma	96843	Immunoblastic lymphoma
Burkitt lymphoma	96873	Burkitt lymphoma
Burkitt lymphoma	98263	Burkitt cell leukemia
Marginal zone lymphoma	96893	Splenic marginal zone lymphoma
Marginal zone lymphoma	96993	Marginal zone lymphoma, NOS
Marginal zone lymphoma	97643	Immunoproliferative small intestinal disease

Cutaneous T-cell lymphoma	97003	Mycosis fungoides
Cutaneous T-cell lymphoma	97013	Sézary syndrome
Cutaneous T-cell lymphoma	97083	Subcutaneous panniculitis-like T-cell lymphoma
Cutaneous T-cell lymphoma	97093	Cutaneous T-cell lymphoma, NOS
Cutaneous T-cell lymphoma	97183	Primary cutaneous CD30+ T-cell disorder
Other mature T/NK cell lymphomas	97023	Peripheral T-cell lymphoma, NOS
Other mature T/NK cell lymphomas	97053	Angioimmunoblastic T-cell lymphoma
Other mature T/NK cell lymphomas	97143	Anaplastic large cell lymphoma
Other mature T/NK cell lymphomas	97163	Hepatosplenic gamma-delta T-cell lymphoma
Other mature T/NK cell lymphomas	97173	Intestinal T-cell lymphoma
Other mature T/NK cell lymphomas	97193	NK/T-cell lymphoma, nasal type
Other mature T/NK cell lymphomas	98273	Adult T-cell leukemia/lymphoma
Other mature T/NK cell lymphomas	98313	T-cell large granular lymphocytic leukemia
Other mature T/NK cell lymphomas	98343	T-cell prolymphocytic leukemia
Other mature T/NK cell lymphomas	99483	Aggressive NK-cell leukemia
Lymphoblastic leukemia/lymphoma	97273	Precursor lymphoblastic lymphoma, NOS
Lymphoblastic leukemia/lymphoma	97283	Precursor B-lymphoblastic lymphoma
Lymphoblastic leukemia/lymphoma	97293	Precursor T-lymphoblastic lymphoma
Lymphoblastic leukemia/lymphoma	98353	Precursor lymphoblastic leukemia, NOS
Lymphoblastic leukemia/lymphoma	98363	Precursor B-lymphoblastic leukemia
Lymphoblastic leukemia/lymphoma	98373	Precursor T-lymphoblastic leukemia
Plasma cell neoplasms	97313	Plasmacytoma, NOS
Plasma cell neoplasms	97323	Multiple myeloma
Plasma cell neoplasms	97333	Plasma cell leukemia
Plasma cell neoplasms	97343	Extramedullary plasmacytoma
B-cell prolymphocytic leukemia	98333	B-cell prolymphocytic leukemia
Hairy cell leukemia	99403	Hairy cell leukemia
Lymphoid leukemia, NOS	98203	Lymphoid leukemia, NOS
Lymphoid leukemia, NOS	98323	Prolymphocytic leukemia, NOS
Leukemia, NOS	98003	Leukemia, NOS
Leukemia, NOS	98013	Acute leukemia, NOS
Leukemia, NOS	98053	Acute biphenotypic leukemia
Leukemia, NOS	98063	Acute mixed phenotype leukemia
Myeloid leukemia, NOS	98603	Myeloid leukemia, NOS
Acute myeloid leukemia	98403	Acute erythroid leukemia
Acute myeloid leukemia	98613	AML, NOS
Acute myeloid leukemia	98663	Acute promyelocytic leukemia
Acute myeloid leukemia	98673	Acute myelomonocytic leukemia
Acute myeloid leukemia	98703	Acute basophilic leukemia
Acute myeloid leukemia	98713	AML with abnormal eosinophils
Acute myeloid leukemia	98723	AML minimal differentiation
Acute myeloid leukemia	98733	AML without maturation
Acute myeloid leukemia	98743	AML with maturation

Acute myeloid leukemia	98913	Acute monocytic leukemia
Acute myeloid leukemia	98953	AML with multilineage dysplasia
Acute myeloid leukemia	98963	AML t(8;21)
Acute myeloid leukemia	98973	AML 11q23 abnormalities
Acute myeloid leukemia	99103	Acute megakaryoblastic leukemia
Acute myeloid leukemia	99203	Therapy-related AML
Acute myeloid leukemia	99303	Myeloid sarcoma
Acute myeloid leukemia	99313	Acute panmyelosis with myelofibrosis
Acute myeloid leukemia	99843	RAEB in transformation
Acute myeloid leukemia	99873	Therapy-related MDS
Myeloproliferative neoplasms	97403	Mastocytoma
Myeloproliferative neoplasms	97413	Malignant mastocytosis
Myeloproliferative neoplasms	97423	Mast cell leukemia
Myeloproliferative neoplasms	98633	Chronic myeloid leukemia
Myeloproliferative neoplasms	98753	CML BCR-ABL1 positive
Myeloproliferative neoplasms	99503	Polycythemia vera
Myeloproliferative neoplasms	99603	MPN, NOS
Myeloproliferative neoplasms	99613	Primary myelofibrosis
Myeloproliferative neoplasms	99623	Essential thrombocythemia
Myeloproliferative neoplasms	99633	Chronic neutrophilic leukemia
Myeloproliferative neoplasms	99643	Hypereosinophilic syndrome
Myelodysplastic syndromes	99803	Refractory anemia
Myelodysplastic syndromes	99823	Refractory anemia with sideroblasts
Myelodysplastic syndromes	99833	Refractory anemia with excess blasts
Myelodysplastic syndromes	99853	Refractory cytopenia with multilineage dysplasia
Myelodysplastic syndromes	99863	MDS with 5q deletion
Myelodysplastic syndromes	99893	MDS, NOS
MDS/MPN overlap	99453	Chronic myelomonocytic leukemia
MDS/MPN overlap	99463	Juvenile myelomonocytic leukemia
MDS/MPN overlap	98763	Atypical CML
MDS/MPN overlap	99753	MDS/MPN, unclassifiable

Supplementary Table S2. Distribution of haematological malignancies diagnosed among adolescents and young adults (AYAs) in Poland between 2000 and 2022, by tumour type, subtype, and sex.

Tumour type	Total, n	Male, n (%)	Female, n (%)	M:F ratio
Lymphoma, NOS	955	546 (57.2)	409 (42.8)	1.33
Non-Hodgkin lymphoma, NOS	687	394 (57.4)	293 (42.6)	1.34
Composite Hodgkin and NHL	13	9 (69.2)	4 (30.8)	2.25
Hodgkin lymphoma, nodular lymphocyte predominance	269	154 (57.2)	115 (42.8)	1.34
Classical Hodgkin lymphoma	9654	4411 (45.7)	5243 (54.3)	0.84
CLL/SLL	432	268 (62.0)	164 (38.0)	1.63
Immunoproliferative diseases	72	32 (44.4)	40 (55.6)	0.80
Mantle cell lymphoma	72	50 (69.4)	22 (30.6)	2.27
Follicular lymphoma	847	419 (49.5)	428 (50.5)	0.98
Diffuse B-cell lymphoma	2089	1179 (56.4)	910 (43.6)	1.30
Burkitt lymphoma	371	281 (75.7)	90 (24.3)	3.12
Marginal zone lymphoma	189	94 (49.7)	95 (50.3)	0.99
Cutaneous T-cell lymphoma	167	96 (57.5)	71 (42.5)	1.35
Other mature T/NK cell lymphomas	589	346 (58.7)	243 (41.3)	1.42
Lymphoblastic leukemia/lymphoma	1815	1157 (63.7)	658 (36.3)	1.76
Plasma cell neoplasms	413	253 (61.3)	160 (38.7)	1.58
Hairy cell leukemia	108	72 (66.7)	36 (33.3)	2.00
Lymphoid leukemia, NOS	103	64 (62.1)	39 (37.9)	1.64
Leukemia, NOS	165	99 (60.0)	66 (40.0)	1.50
Myeloid leukemia, NOS	105	69 (65.7)	36 (34.3)	1.92
Acute myeloid leukemia	2150	1140 (53.0)	1010 (47.0)	1.13
Myeloproliferative neoplasms	1684	947 (56.2)	737 (43.8)	1.29
Myelodysplastic syndromes	103	42 (40.8)	61 (59.2)	0.69
MDS/MPN overlap	35	13 (37.1)	22 (62.9)	0.59

Note: Myeloproliferative neoplasms, myelodysplastic syndromes, and MDS/MPN overlap neoplasms were excluded from trend and projection analyses due to limited comparability of registry data from the pre-PROH period.
